# Leveraging innovation technologies to respond to malaria: a systematized literature review of emerging technologies

**DOI:** 10.1186/s12936-023-04454-0

**Published:** 2023-02-03

**Authors:** Moredreck Chibi, William Wasswa, Chipo Ngongoni, Ebenezer Baba, Akpaka Kalu

**Affiliations:** 1 World Health Organization Africa Region, Brazzaville, Republic of Congo; 2 Tropical and Vector Borne Diseases, Universal Health Coverage/Communicable and Non Communicable Disease Cluster, World Health Organization Africa Region, Brazzaville, Republic of Congo

**Keywords:** Malaria, Emerging technologies, eHealth, mHealth, Innovation

## Abstract

**Background:**

In 2019, an estimated 409,000 people died of malaria and most of them were young children in sub-Saharan Africa. In a bid to combat malaria epidemics, several technological innovations that have contributed significantly to malaria response have been developed across the world. This paper presents a systematized review and identifies key technological innovations that have been developed worldwide targeting different areas of the malaria response, which include surveillance, microplanning, prevention, diagnosis and management.

**Methods:**

A systematized literature review which involved a structured search of the malaria technological innovations followed by a quantitative and narrative description and synthesis of the innovations was carried out. The malaria technological innovations were electronically retrieved from scientific databases that include PubMed, Google Scholar, Scopus, IEEE and Science Direct. Additional innovations were found across grey sources such as the Google Play Store, Apple App Store and cooperate websites. This was done using keywords pertaining to different malaria response areas combined with the words “innovation or technology” in a search query. The search was conducted between July 2021 and December 2021. Drugs, vaccines, social programmes, and apps in non-English were excluded. The quality of technological innovations included was based on reported impact and an exclusion criterion set by the authors.

**Results:**

Out of over 1000 malaria innovations and programmes, only 650 key malaria technological innovations were considered for further review. There were web-based innovations (34%), mobile-based applications (28%), diagnostic tools and devices (25%), and drone-based technologies (13%.

**Discussion and conclusion:**

This study was undertaken to unveil impactful and contextually relevant malaria innovations that can be adapted in Africa. This was in response to the existing knowledge gap about the comprehensive technological landscape for malaria response. The paper provides information that countries and key malaria control stakeholders can leverage with regards to adopting some of these technologies as part of the malaria response in their respective countries.

The paper has also highlighted key drivers including infrastructural requirements to foster development and scaling up of innovations. In order to stimulate development of innovations in Africa, countries should prioritize investment in infrastructure for information and communication technologies and also drone technologies. These should be accompanied by the right policies and incentive frameworks.

## Background

In sub-Saharan Africa, malaria is the leading cause of death for children under 5. It has been reported that malaria infection during pregnancy increases the risk of maternal mortality and neonatal mortality [1]. According to the World Health Organization (WHO), there were 229 million cases of malaria in 2019 compared to 228 million cases in 2018. The estimated number of malaria deaths stood at 409,000 in 2019, compared with 411,000 deaths in 2018. Children under 5 years of age are the most vulnerable group affected by malaria and in 2019 they accounted for 67% (274,000) of all malaria deaths worldwide. The WHO African Region continues to carry a disproportionately high share of the global malaria burden. In 2019, the region was home to 94% of all malaria cases and deaths with six countries accounting for approximately half of all malaria deaths worldwide: Nigeria (23%), the Democratic Republic of the Congo (11%), United Republic of Tanzania (5%), Burkina Faso (4%), Mozambique (4%) and Niger (4%) [2].

Knowledge, learning and innovation are key to addressing, minimizing and tackling these disparities. One example of this is the knowledge hub developed by WHO called MAGICapp which aims to give living evidence and resources for tackling malaria interventions. It contains all official WHO recommendations for malaria prevention (vector control and preventive chemotherapies) and case management (diagnosis and treatment). The resources serve as a guide on the strategic use of information to drive impact, surveillance, monitoring and evaluation; operational manuals, handbooks, and frameworks; and a glossary of key terms and definitions. So, this paper aligns with identifying and adding discourse into the importance of reviews especially from a technological perspective.

To understand the advances in malaria services, various scholars have undertaken reviews across vast thematic areas of malaria interventions. In a quest to inform policy, Garner et al. [3] conducted an analysis of why Cochrane Reviews are important in malaria interventions. They noted that it is important for researchers to collaborate across regions and in understanding new preventive interventions. Their aim was to inform policymakers to understand the importance of reviews in identification of trends that are occurring in malaria interventions. Other aspects that have been looked at through reviews are the costs and cost-effectiveness aligned with malaria control interventions. White et al. [4] looked at interventions from studies published between 2000 and 2010 looking at the role of infection detection technologies for malaria elimination and eradication and the costs related to them in order to assess how accessible interventions are across regions. More recently, Conteh et al. [5] also carried on with assessing the unit cost and cost-effectiveness of malaria control during the period of January 1, 2005, and August 31, 2018. The aim was to see how resource allocation can be planned proactively according to costs, though they did highlight that care in methodological and reporting standards is required to enhance data transferability.

In a bid to combat malaria epidemic, several technological innovations have been developed all over the world that have contributed significantly to malaria response. Adeola et al. [6] reviewed the use of spatial technology for malaria epidemiology in South Africa between 1930 and 2013. The focus was on the use of statistical and mathematical models as well as geographic information science (GIS) and remote sensing (RS) technology for malaria research to create a robust malaria warning system. The mathematical modelling is also aligned with agent-based modelling which Smith et al. [7] highlighted through their analysis of 90 articles published between 1998 and May 2018 characterizing agent-based models (ABMs) relevant to malaria transmission. The aim was to provide an overview of key approaches utilized in malaria prevention. Such technologies feed into modelling sites and interventions to project various outcomes. From a platform centric perspective, Vasiman et al. [8] analysed how different mobile phone devices and handheld microscopes work as diagnostic platforms for malaria in low-resource settings. Malaria diagnostics tests and methods have also been reviewed as being key in the successful control and elimination programmes [9]. Mobile health has been found to play a key role in supporting health workers in the diagnosis and treatment of malaria in sub-Saharan Africa [10].

To add to this discourse, this paper presents a holistic systematized review of key technological innovations that have been developed worldwide targeting different areas of the malaria response, which include surveillance, microplanning, prevention, diagnosis, and management. A systematized review was utilized in this study as data sources that included unconventional grey sources was utilized and the review gravitated more towards being narrative with tabular accompaniments as compared to the systematic literature reviews that are less narrative [11]. The study was undertaken with the view to provide African countries and key stakeholders with information relating to technologies that can be adapted in their different contexts as they strengthen malaria response strategies.

## Methods

### Scientific databases literature search

**Fig. 1:**
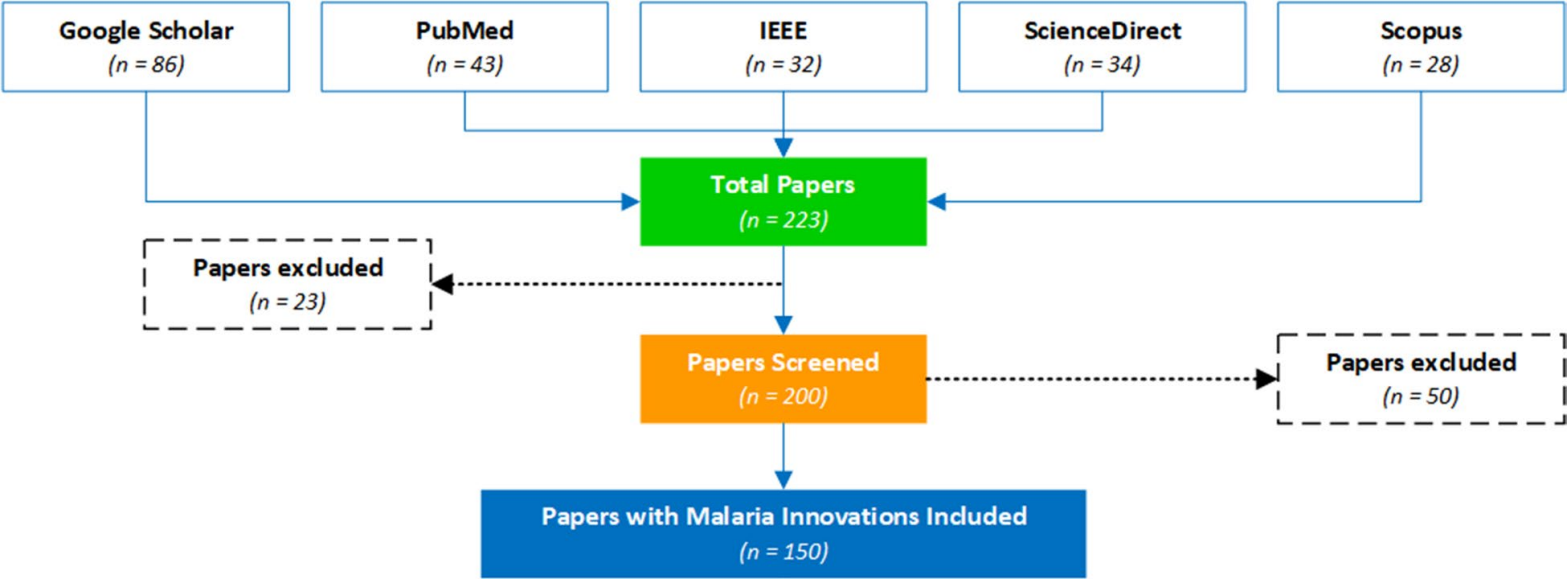
PRISMA flow chart for the malaria innovations literature search

This study adopted a systematic search strategy to identify the publications with innovations related to malaria surveillance, microplanning, prevention, diagnosis, and management from 5 scientific databases (PubMed, Google Scholar, Scopus, IEEE and Science Direct). The keywords used were malaria surveillance, microplanning, prevention, diagnosis and management combined with the words “innovations” or “technologies” in a search query. Innovations deemed not relevant to the scope of this research by the authors include drugs, vaccines, social programmes. Only papers reporting design, implementation or evaluation of malaria technological innovations were considered in this paper. The process was shown in Fig. 1. The quality of technological innovations included was based on reported impact and judgement by the authors.

### Search through technology platforms e.g., google play store and apple app store

**Fig. 2:**
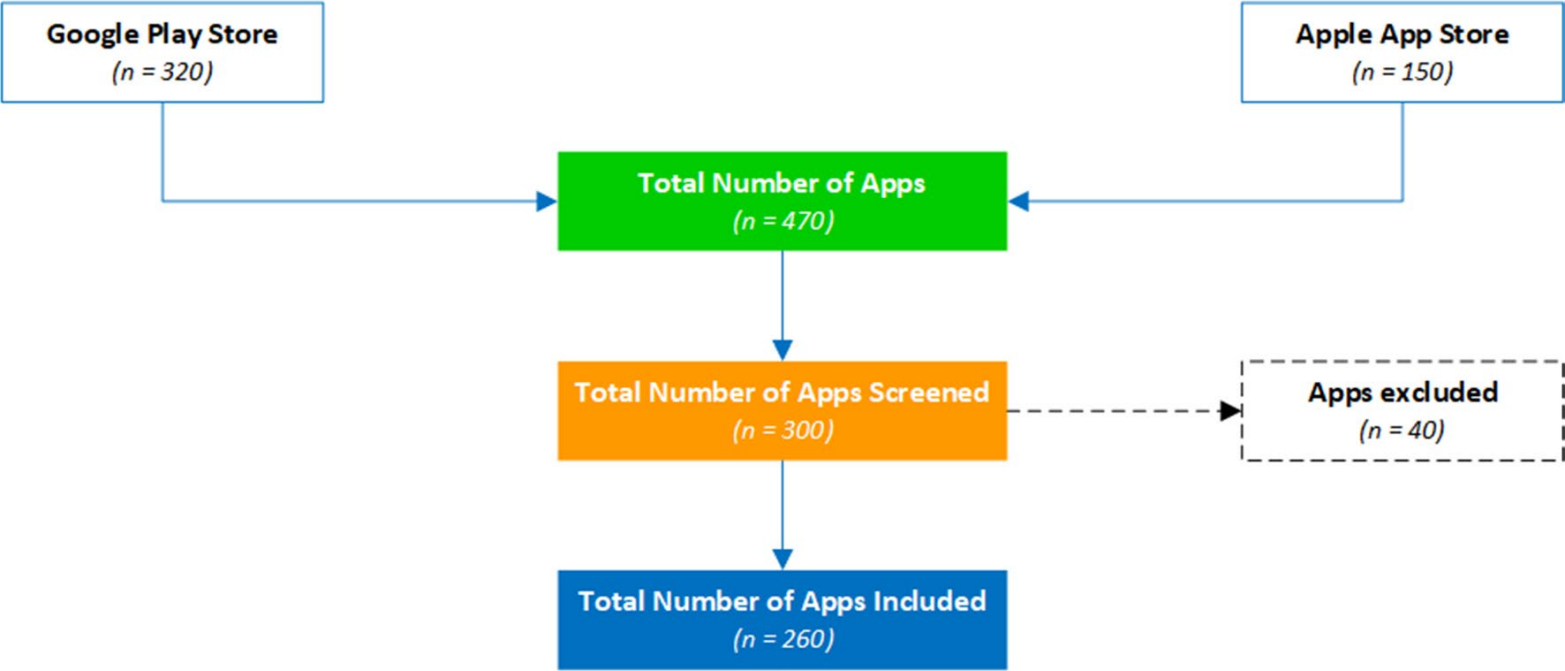
PRISMA flow chart for the mobile apps

This study also adopted a systematic search strategy to identify the mobile apps related to malaria surveillance, microplanning, prevention, diagnosis and management available in the Google Play and Apple App stores. Keywords such as malaria surveillance, microplanning, prevention, diagnosis, and management were used in the search. The search was conducted between July 2021 and December 2021. The applications had to have a description, be in English, have 1000 + installs and reviews to be included in the analysis. The applications that did not meet these criteria were excluded. The core research question was: *What mobile-based innovations are available for malaria interventions that can be adopted by the countries in the WHO Africa region for use across the continuum of the malaria response*? The resultant apps considered for this study were 260 as shown in Fig. 2.

### Web search using a custom web-content mining algorithm

A custom web-content mining algorithm was also developed to search for malaria innovations and technologies published on different cooperate organizational websites, social media channels like twitter, and media channels like legit news websites like CNN. These technological innovations were collated between July 2021 and December 2021. The innovation name, description, Intellectual Property owner, web link to the innovation and geographical location were collated. Innovations that did not have functional and tested prototypes and were not related to addressing malaria interventions were excluded. The number of innovations surpassed 1000 however after screening, only 240 key technological innovations were selected that best fit the selection criteria.

A total of 650 malaria innovations (260 from Google play and Apple App store, 150 from scientific databases and 240 from web content mining) were considered for detailed review.

## Results

The review has identified innovations for malaria in the following technological thematic areas; web-based innovations (34%), mobile-based applications (28%), diagnostic tools and other devices (25%), and drone-based technologies (13%).

### Web-based innovations

The web-based technologies include GIS systems [12]. An example is the Malaria Atlas Project (MAP), developed at the Telethon Kids Institute, Perth, Western Australia. MAP is a web platform that displays time aware raster and survey point data for malaria incidence, endemicity, and mosquito distribution. MAP has been designated as a WHO Collaborating Centre in Geospatial Disease Modelling. The impact of the Atlas Project has been validated in Sokoto Nigeria by Nakakana et al. [13]. The study concluded that the prevalence of malaria and its transmission intensity in Sokoto are similar to the Malaria Atlas Project predictions for the area and that is essential in modellings various aspects of malaria control planning purposes.

Other innovations like malariaAtlas which is an open-access R-interface on the Malaria Atlas Project, collates malariometric data, providing reproducible means of accessing such data within a freely available and commonly used statistical software environment [14]. A team from the University of Queensland developed a GIS-based spatial decision support system (SDSS) used to automatically locate and map the distribution of confirmed malaria cases, rapidly classify active transmission foci, and guide targeted responses in elimination zones. This has been implemented and evaluated in the Solomon Islands and Vanuatu in a study by Kelly et al. [15] and 82.5% of confirmed malaria cases were automatically geo-referenced and mapped at the household level, with 100% of remaining cases geo-referenced at a village level using the system. The GIS-based spatial decision support system has also been implemented in other countries like Vietnam. In Korea, the Malaria Vulnerability Map Mobile System which consists of a system database construction, malaria risk calculation function, visual expression function, and website and mobile application has been developed for use in Incheon [16]. The Malaria Decision Analysis Support Tool (MDAST) project promotes evidence-based, multi-sectoral malaria control policy-making in Kenya, Tanzania, and Uganda, serving as a pilot for such a programme in other malaria-prone countries [17].

In Zanzibar, the Malaria Case Notification (MCN) System was developed and the performance evaluation of the tool by Khandekar [18] showed that while a surveillance system can automate data collection and reporting, its performance will still rely heavily on health worker performance, community acceptance, and infrastructure within a country. A study by Mody et al. [19] showed that the use of telemedicine and e-health technologies shows promise for the remote diagnosis of malaria and hence several systems been developed. ProMED Mail (PMM) is an open and free to use, global, e-health based surveillance system from the International Society for Infectious Diseases with several use cases for malaria [20, 21]. The Epidemic Prognosis Incorporating Disease and Environmental Monitoring for Integrated Assessment (EPIDEMIA) computer system was designed and implemented to integrate disease surveillance with environmental monitoring in support of operational malaria forecasting in the Amhara region of Ethiopia [22]. Table 1 summarizes some of the technologies.

**Table 1:** Examples of web-based technologies

No	Name	Description
1	Malaria Atlas Project (MAP)	Disseminate free, accurate and up-to-date information on malaria and associated topics, organized on a geographical basis
2	MalariaAtlas	Open-access R-interface to malariometric data, providing access to such data within a freely available and commonly used statistical software environment
3	Spatial decision support system (SDSS)	Automatically locate and map distribution of malaria cases, classify active transmission foci, and guide targeted responses
4	Malaria Vulnerability Map Mobile System (MVMMS)	A malaria surveillance system to automate data collection and reporting
5	Malaria Decision Analysis Support Tool (MDAST)	Promotes evidence-based, multi-sectoral malaria control policy-making
6	Malaria Case Notification (MCN) System	Automate data collection and incidence reporting

### Mobile applications-based technologies

This study has also revealed that several mobile-based malaria innovations have been developed which include smart mobile apps, Short Message Service (SMS) based apps and Unstructured Supplementary Service Data (USSD) based applications for use across the continuum of the malaria response. In India the Mobile-based Surveillance Quest using IT (MoSQuIT) is being used to automate and streamline malaria surveillance for all stakeholders involved, from health workers in rural India to medical officers and public health decision-makers. Malaria Epidemic Early Detection System (MEEDS) is a groundbreaking mHealth system used in Zanzibar by health facilities to report new malaria cases through mobile phones. Coconut Surveillance is an open-source mobile software application designed by malaria experts specifically for malaria control and elimination and it has become an essential tool for the Zanzibar Malaria Elimination Programme [23]. The SMS for Life initiative is a ‘public-private’ project that harnesses everyday technology to eliminate stock-outs and improve access to essential medicines in sub-Saharan Africa with a health focus on malaria and other vector borne diseases. This has been implemented and evaluated in Tanzania [24]. In Mozambique Community Health Workers (CHWs) use inSCALE CommCare tool for decision support, immediate feedback and multimedia audio and images to improve adherence to protocols.

Additional surveillance apps include the likes of the DHS mobile app for Malaria Indicator Surveys and Solution for Community Health-workers (SOCH) mobile app is a comprehensive mobile application tool for disease surveillance, workforce management and supply chain management for malaria elimination [25]. The National Malaria Case-Based Reporting App (MCBR) is a mobile phone application for malaria case-based reporting to advance malaria surveillance in Myanmar [26]. Mobile apps have also been used to support distribution of medicines like the Net4Schs App, an android application that is used for data capturing, processing and reporting on School Long-lasting insecticidal nets (LLINs) distribution activities. Apps have also been developed to support malaria screening and diagnosis for example the NLM Malaria Screener is a diagnostic app that assists users in the diagnosis of malaria and in the monitoring of malaria patients. This has been validated in several studies and it is reported that it makes the screening process faster, more consistent, and less dependent on human expertise [27]. Additional diagnostic apps include the Malaria System MicroApp which is a mobile device-based tool for malaria diagnosis [28], the Malaria Hero app is a web based mobile app for diagnosis of malaria, and LifeLens is a smartphone app that can detect malaria. Some key technologies are summarized in Table 2.

**Table 2:** Examples of mobile application- based technologies

No	Name	Description
1	Mobile-based Surveillance Quest using IT (MoSQuIT)	Automate and streamline malaria surveillance among stakeholders involved
2	Malaria Epidemic Early Detection System (MEEDS)	Reporting new malaria cases through mobile phones
3	Coconut Surveillance	An open-source mobile software application designed by malaria experts specifically for malaria control and elimination
4	inSCALE CommCare tool	For decision support, immediate feedback and multimedia audio and images to improve adherence to protocols
5	Malaria System MicroApp	Mobile device-based tool for malaria diagnosis
6	Net4Schs App	Mobile App used for data capturing, processing and reporting on School Long-lasting insecticidal nets (LLINs) distribution activities

Other notable mobile apps that have also been used in malaria management include CommCare’s usage in in Mozambique for integrated community case management in the remote communities. This has been reported to strengthen Community-Based Health [29]. Another app, FeverTracker, has been used for malaria surveillance and patient information management in India. There has also been a number of educational and knowledge base apps. These are the likes of Malaria Consultant, a mobile application designed to educate individuals on malaria and its prevention; the WHO Malaria toolkit App that brings together the content of the latest world malaria report and of the consolidated WHO Guidelines for malaria. This includes operational manuals for carrying out malaria interventions and other technical documents in one easy to navigate resource. Another interesting area where mobile apps have been used is in malaria prevention and such apps include those that scare away mosquitoes using high frequency sounds, and these include Anti Mosquito Repellent Sound App.

### Drone-based technologies

This review has revealed that drone technologies can greatly help in malaria control programmes. The drones can be used in developing genetically-based vector control tools [30], delivering massive aerial spraying to kill mosquito larvae [31], identifying mosquito larvae sites using aerial imaging [32] and in delivering drugs and vaccines [33]. Anti-malaria drones have been widely used to spray biological insecticides in rice fields and swamps to reduce the emerging mosquito populations. This has been successful in Kenya, Tanzania, India, Rwanda and Zanzibar. In Zanzibar, the Agras MG-1S drones were used to spray 10 L of a biodegradable agent called Aquatain; a chemical that has been used to cover drinking water basins. Drones have also been used to collect data to identify mosquito breeding sites so that the larvae can be controlled, reducing the number of adult mosquitoes able to spread malaria. For example in Malawi and near Lake Victoria the DJI Phantom low-cost drones are being used to survey and find mosquito breeding grounds. A new trial using ‘gene drive’ technology is currently taking place in Burkina Faso where the trial will see the release of genetically modified mosquitoes in an attempt to wipe out the female carriers of the disease [34].

### Diagnostic tools including other devices developed for malaria interventions

Devices that have been developed to respond to malaria include the SolarMal device, a solar-powered mosquito trapper being piloted in Kenya [35]. The Solar Powered Mosquito Trap (SMOT) is baited with a synthetic odor blend that mimics human odor to lure host-seeking malaria mosquitoes. Other devices such as the ThermaCell Patio Shield Mosquito Repellants developed by ThermaCell are shield lanterns that repel mosquitoes by creating a 15-foot zone of protection. Several devices have also been developed to improve malaria diagnosis and these include the Nanomal DNA analyzer a simple, rapid and affordable point-of-care (POC) handheld diagnostic nanotechnology device to confirm malaria diagnosis and detect drug resistance in malaria parasites in minutes and at the patient’s side, by analysis of mutations in malaria DNA using a range of proven nanotechnologies. Medication Events Monitoring Device (MEMS) have also been greatly used to monitor medication adherence to malaria drugs [36]. Malaria Rapid Diagnostic Tests (RDTs), sometimes called dipsticks or Malaria Rapid Diagnostic Devices (MRDDS), are simple immunochromatographic tests that identify specific antigens of malaria parasites in whole or peripheral blood. They are categorized into dipstick, cassette or hybrids. Dipstick RDTs are cheap and readily available on market [37]. An example is the OptiMAL dipstick [38]. Cassette RDTS are complex and require much time for results to be read but are much safer to use.

## Discussion

This research has culminated into insightful conclusions from the systematized review of the malaria technological Innovations and has been the foundation of the collated database that can be accessed via the WHO AFRO marketplace platform. This is a platform that has been developed to showcase various technologies and innovations that can be applied for different disease areas. This focused on technologies relevant for malaria response. The identified intervention technologies and focus areas provide ways of identifying key leverage points in strengthening the health systems and making tangible impact towards various mandates to fight the scourge of malaria. More importantly highlighting these trends empowers innovators and policy makers on the continent to make informed decisions on applying frugal design to develop affordable, locally manufactured, functional and sustainable innovations fit for the African continent. Furthermore, the marketplace platform provides implementation insights to African nations on the adoption of some of the technological innovations from this study.

**Fig. 3:**
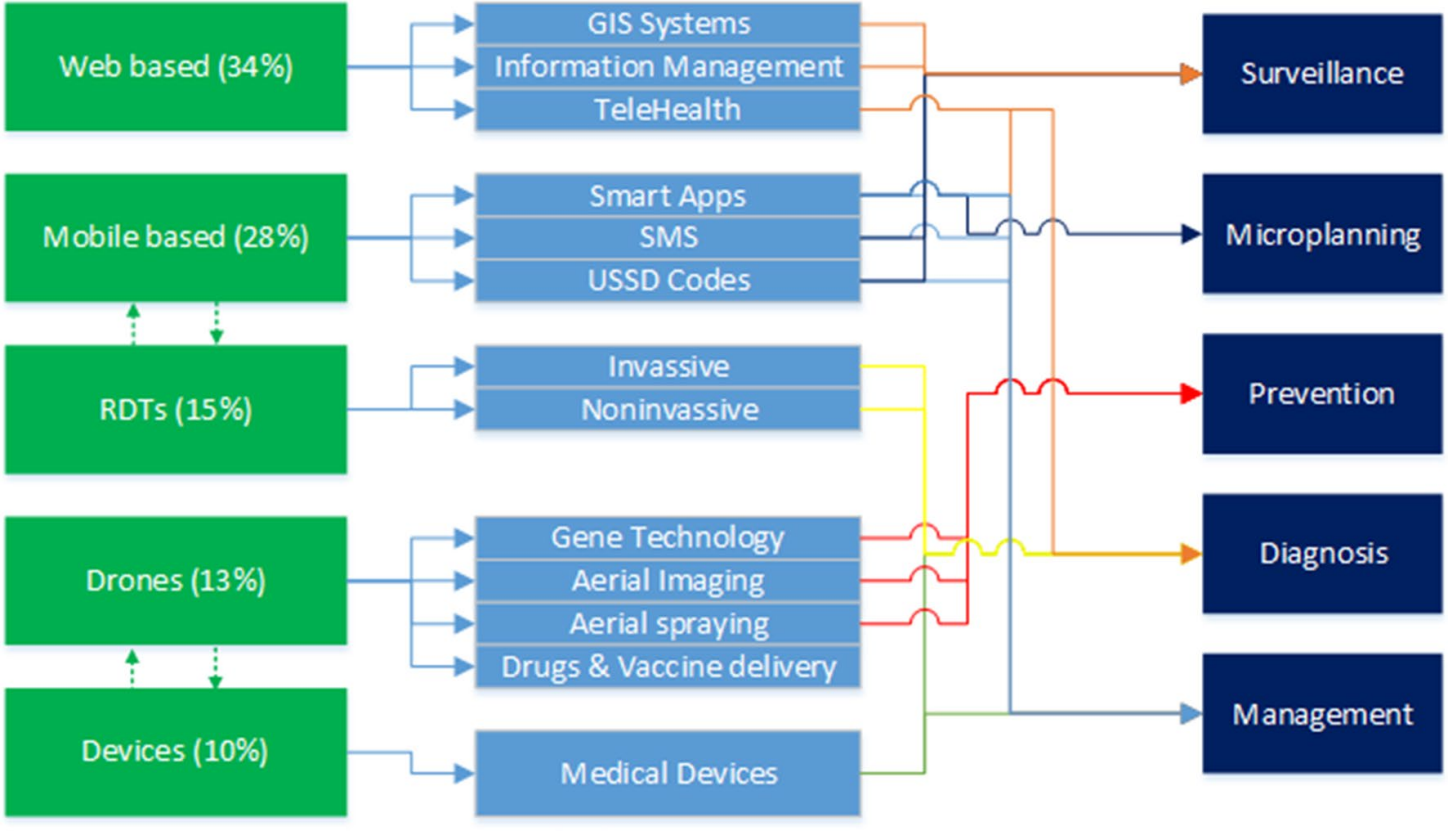
Analysis of the innovations by category, application and target outcome

The review has highlighted that mobile applications are a vital component of malaria response programmes and are increasingly being used along the different response areas, such as surveillance (malaria data capturing apps like Coconut Surveillance and DHS mobile app), microplanning (drug delivery and distribution management apps like Net4Schs App), prevention (mosquito repelling like Anti Mosquito Repellent Sound App), diagnosis (AI driven slide analysis apps like LifeLens and Malaria Screener), management (telehealth like the Malaria Consultant) and the provision of support for health services [decision support like the solution for Community Health-workers (SOCH) app] as outlined in Fig. 3. Their impact has been validated in several studies [27, 39].

In 2019, 93% of the global population was covered by a mobile broadband signal. In Sub-Saharan Africa, 3G coverage expanded to 75% compared to 63% in 2017, while 4G doubled to nearly 50% compared to 2017 [40]. This implies that mobile solutions can substantially mitigate many of the health system limitations prevalent mostly in African countries where malaria is endemic. A substantial number of mobile applications have been developed for surveillance of malaria control programs in Africa such as inSCALE (Mozambique), Coconut Surveillance (Zanzibar), CommCare (Senegal) and DHIS2 (Zimbabwe, and South Africa). This shows that mobile-based apps give a larger footprint and a high level of agility to malaria response. Nevertheless, limited connectivity and erratic energy supplies have been key factors affecting the levels of adoption and some apps have been reported to have a high level of complexity. This has also been reported in other studies [41, 42].

Moreover, it has been noted that most of these apps are independent with limited capability for interoperability. Hence there is a need to develop open standards for mobile technologies for malaria control. For example, surveillance applications should be able to have geolocation capabilities and use exiting open-source platforms like OpenStreetMap, OpenDataKit & OpenMapKit; work online and offline mode to enable usage in resource constraints areas, ease of use to enable usage with little or no training and should support different languages including local languages. This calls for more research and implementation of natural language processing frameworks for use in mobile apps in Africa, which can assist with data analytics as well. Furthermore, aligning app development with standards such as the Fast Healthcare Interoperability Resources (FHIR) which facilitate interoperability between legacy health care systems and technology is important.

Superseding technological interoperability, there needs to be platform integration and overall visibility particularly on innovations that target malaria diagnosis, surveillance and management. However, it should be noted that systemically there has been launching of different applications for different malaria interventions which may confuse the public in terms of usage. Therefore, a single application or platform integrating several services such as Coconut Surveillance and owned and managed by a reputable malaria organization or the ministries of health may benefit citizens by allowing them to access services from a single and trusted application. Misinformation and misdiagnosis from publicly available medical apps is a health threat to the public as reported by [43].

Most of the reviewed web systems depend on data or are used to collect large amounts of malaria data to support decision-making. Hence a need for national malaria control and elimination information systems that can utilize regional and global structures, prioritizing cross-border intelligence sharing information regarding disease transmission hotspots, outbreaks, and human movement. Such systems can also be very useful in responding to pandemics like COVID-19 and other infectious outbreaks. There is also a need to have malaria related data centrally stored and managed by the Ministry of Health or malaria control programmes to guide decision-making at all levels of malaria response among the different stakeholders. Hospitals and clinics have also developed standalone patient information management systems in addition to the national health information management systems like OpenMRS and DHIS2. However, there is no communication between the different patient’s information management systems hence a need for development of open data standard driven systems and APIs to enforce interoperability among health systems in Africa. An effective information system must receive data from other sources, process it and send it back to other systems being used in malaria programme, particularly at the community level.

In malaria control, larval source management is very difficult to archive in rural areas due to perceived difficulties in identifying target areas [44]. Drones can capture extremely detailed images of the landscape, opening the possibility of replacing the time-consuming hunt for mosquito larvae on the ground with identifying habitat through aerial imagery. The review has shown that this has been used in several countries for example in Malawi and near Lake Victoria using DJI Phantom; low-cost drones that survey wilderness to find mosquito breeding grounds using Geospatial technology. Geospatial technology is rapidly evolving and now can be archived using remotely sensed data [45]. In Zanzibar, drones have been used to spray rice fields with a thin, non-toxic film as a strategy to eliminate mosquitoes. The review has shown that drones are a possible solution in malaria control programmes as also indicated in other studies [45, 46]. The review also showed that rapid diagnostics tools offer fast turnaround services while circumventing obstacles faced when using microscopy in peripheral health care settings, including cost of equipment, reagents, and the need for electricity and skilled personnel [47].

## Conclusion

This study has reviewed key emerging technologies used in malaria control programmes. The review revealed various technological applications that have been developed in response to malaria including surveillance, microplanning, prevention, diagnosis and management. Although breakthrough innovative platforms have been made available, one key challenge remained, which is lack of integration of key end-to-end components and functionalities to facilitate effective and efficient malaria response and to reduce fragmentation.

The review has also revealed several stakeholders in malaria control hence a need for mechanisms that promote the exchange of evidence between scientific, policy, and programme management communities for analysing the potential outcomes of the different malaria control strategies and interventions. In many malaria-endemic areas in Africa, the communication gap between policy makers, health workers, and patients is a significant barrier to efficient malaria control.

Furthermore, artificial intelligence (AI) has been widely used in the reviewed technological innovations, however there is an urgent need to provide reliable datasets, develop local AI expertise among WHO African member states, implement data protection and privacy acts; and put in place health innovation clusters to bring the different stakeholders together to develop and adopt appropriate technologies to solve the intended challenges.

### Limitations of this work and future prospects

The main limitation of this work was that some applications were overlapping among the response areas and hence the decision to place an innovation under a given category was based on the judgement of the authors. Another limitation is the fact that this work is not aimed at analysing the total landscape of all malaria innovations. Only those that met the inclusion criteria and deemed relevant by the authors were included hence some innovations might not have been captured but we will be subjected to continuous update on the global database for malaria innovations at https://innov.afro.who.int/emerging-technological-innovations/7-malaria-innovations. Future research can focus on reviewing the technologies that are open source dedicated to malaria, and publishing findings that can be used by medical practitioners, application developers, and governments to collaborate in the process of containing the spread of malaria.

## Data Availability

The data used in this report is available to readers.
